# The epithelial barrier: a Janus-faced regulator in allergic airway diseases from defense to inflammation

**DOI:** 10.3389/falgy.2025.1693611

**Published:** 2025-11-06

**Authors:** Youwei Bao, Qi Chen, Binbin Shi, Xinhua Zhu

**Affiliations:** Department of Otorhinolaryngology Head and Neck Surgery, The Second Affiliated Hospital of Nanchang University, Jiangxi Medical College, Nanchang University, Nanchang, Jiangxi, China

**Keywords:** epithelial barrier, allergic respiratory diseases, immune defense, inflammatory amplification, immunomodulation

## Abstract

**Background:**

The epithelial barrier serves as the body's first line of defense between the host immune system and the external environment. Evidence confirms that epithelial barrier damage is an initial event in the pathogenesis of allergic respiratory diseases, during which the barrier exhibits a dual “Janus-faced” role.

**Methods:**

This review synthesizes current literature to explore the molecular and cellular mechanisms underlying epithelial barrier dysfunction, including dysregulation of tight junctions, aberrant immune signaling, and release of pro-inflammatory alarmins. We also evaluate contemporary diagnostic technologies for assessing the epithelial barrier and analyze current therapeutic strategies aimed at its restoration.

**Results:**

An intact respiratory epithelial barrier effectively defends against allergens and pathogens. When compromised, it exacerbates inflammatory responses through the release of alarmins. Advances in omics-based profiling and advanced imaging now enable precise assessment of barrier integrity. Therapeutically, innovative strategies—including immunomodulators, biologics, and novel agents targeting epithelial repair pathways—offer promising avenues for restoring barrier function and controlling inflammation.

**Conclusion:**

Real-time and effective diagnosis of epithelial barrier integrity, coupled with therapeutic strategies targeting the barrier, are pivotal for achieving long-term disease control in asthma, allergic rhinitis, and related conditions. Future research should focus on barrier-centric integrated approaches to bridge fundamental scientific discoveries with clinical applications.

## 1 Introduction

Allergic respiratory diseases, particularly asthma and allergic rhinitis, represent a growing global health challenge, affecting hundreds of millions of individuals and imposing a substantial socioeconomic burden by impairing work productivity, academic performance, and quality of life (1–3). Traditional research on allergic diseases has focused on the dysregulated T helper 2 (Th2)-polarized immune response, extensively studying its cellular differentiation processes and therapeutic interventions. This paradigm has effectively explained the roles of IgE, mast cells, eosinophils, and specific cytokines in driving allergic inflammation and symptoms. However, the initial mechanisms leading to sensitization—specifically, damage to the epithelial barrier—and its role in determining disease persistence and severity remain a major focus of current research.

As the critical interface between the host's internal milieu and the external environment, the airway epithelial barrier constitutes the first line of defense against inhaled allergens, pathogens, and pollutants. An intact barrier effectively prevents allergen penetration and promotes immune tolerance, whereas a compromised barrier facilitates allergen translocation and initiates inflammatory cascades (4–7). This dual functionality casts the epithelial barrier in the role of a “Janus-faced regulator” in allergic airway diseases—acting as both a defender of homeostasis and, when impaired, an amplifier of pathology. The mechanisms underlying this duality are multifaceted, involving disruption of tight junction complexes (claudins, occludin), dysregulated crosstalk with innate and adaptive immune cells, and the release of epithelial-derived cytokines known as “alarmins.” These alarmins, such as TSLP, IL-25, and IL-33, act as potent initiators and amplifiers of type 2 inflammation by activating group 2 innate lymphoid cells (ILC2s) and Th2 lymphocytes, establishing a self-perpetuating cycle of inflammation and barrier damage (8, 9). Despite this significant conceptual advancement, a comprehensive synthesis of how barrier dysfunction translates across the entire spectrum of disease initiation, progression, and treatment is still required.

This review aims to bridge this gap by providing a systematic analysis of the pivotal role of the epithelial barrier in allergic respiratory diseases, underscoring the research value in this field. We will delve into its fundamental structure and function, the pathophysiological mechanisms by which it contributes to disease, and the epidemiological and environmental factors influencing its integrity. Furthermore, we will explore cutting-edge diagnostic technologies for assessing barrier function and evaluate innovative therapeutic strategies aimed at barrier restoration and immunomodulation. Finally, we will address current controversies and future research directions, emphasizing the translational potential of targeting the epithelial barrier to achieve long-term disease control and improve patient outcomes.

## 2 Methods

### 2.1 Literature search strategy

A systematic literature search was conducted to identify all relevant publications concerning the role of the epithelial barrier in allergic respiratory diseases. The electronic databases PubMed, Web of Science, and Scopus were comprehensively searched from January 2000 to September 2025. The search strategy was designed by combining keywords and Medical Subject Headings (MeSH) terms related to four core conceptual domains:Epithelial Barrier: (“epithelial barrier” OR “airway epithelium” OR “mucosal barrier” OR “tight junction” OR Claudin OR Occludin OR “ZO-1”); Allergic Diseases: (“asthma” OR “allergic rhinitis” OR “allergic airway disease” OR “atopy”); Key Mechanisms: (“alarmin” OR “TSLP” OR “IL-25” OR “IL-33” OR “ILC2” OR “type 2 inflammation”); Interventions/Assessments: (“diagnos” OR “therap” OR “treatment” OR “biologic” OR “immunotherap”). These terms were combined using the Boolean operators “AND” and “OR” to maximize the retrieval of relevant studies. Additionally, the reference lists of key review articles and eligible primary studies were manually screened to identify other pertinent publications (10–14).

### 2.2 Eligibility criteria

Studies were selected based on the following pre-defined criteria:

Population/Subjects: Studies involving humans (patients or primary cells) or animal/models of allergic asthma and allergic rhinitis.
Intervention/Exposure: Not applicable in the conventional interventional sense; however, the review focused on studies investigating epithelial barrier structure, function, dysfunction, and its interaction with the immune system.
Comparator: Studies comparing healthy vs. diseased states, or assessing the effects of different treatments on barrier function.
Outcomes: Primary outcomes of interest included measures of epithelial barrier integrity (e.g., transepithelial electrical resistance, tight junction protein expression), levels of epithelial-derived cytokines, immune cell activation, and clinical correlates of disease severity.
Study Design: Original research articles (including *in vitro*, *in vivo*, and clinical studies) and high-quality systematic reviews published in English were included. Editorials, conference abstracts, and non-English publications were excluded.

### 2.3 Study selection and data extraction

The study selection process adhered to the Preferred Reporting Items for Systematic Reviews and Meta-Analyses (PRISMA) guidelines. After duplicate removal, two authors independently screened the titles and abstracts of all retrieved records against the eligibility criteria. The full texts of potentially relevant articles were subsequently assessed for final inclusion. Any discrepancies between the reviewers were resolved through discussion or by consultation with a third author.

A standardized data extraction form was used to collect key information from the included studies. Extracted data included: first author, publication year, study type (clinical trial, animal study, *in vitro* experiment), subject/cell model, primary interventions or exposures, methods for assessing barrier function, key findings related to the epithelial barrier, and main conclusions.

### 2.4 Data synthesis and analysis

**Figure 1:**
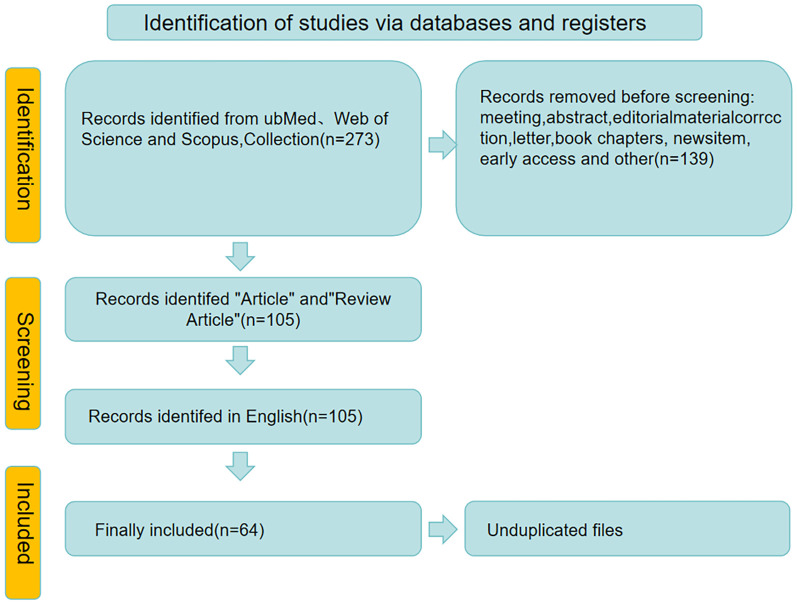
Retrieval and screening flow chart.

Given the narrative nature of this review and the significant methodological heterogeneity of the included studies (encompassing diverse approaches from molecular biology to clinical trials), conducting a formal meta-analysis was not feasible. Therefore, a thematic synthesis approach was adopted. The extracted data were organized and synthesized into coherent thematic sections to provide a comprehensive and critical overview of the current state of knowledge, as presented in this manuscript. The study selection process is summarized in a PRISMA flow diagram (Figure 1).

## 3 Results

### 3.1 Structural and functional characteristics of the epithelial barrier

The airway epithelium, predominantly a pseudostratified ciliated columnar epithelium, is uniquely adapted to its anatomical location. Its structural and functional integrity is maintained by a complex network of intercellular junctions and is supported by specialized clearance mechanisms (15, 16).

#### 3.1.1 The physical barrier: intercellular junctions

**Figure 2:**
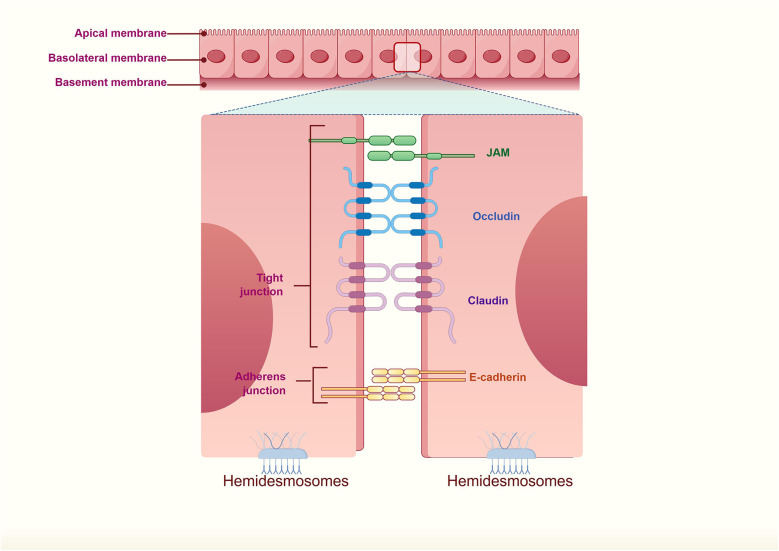
The physical barrier of epithelial cells: intercellular junctions.

The fundamental physical barrier is formed by a continuous band of intercellular junctions. Tight junctions (TJs), situated at the most apical region of the lateral membrane, represent the paramount structures governing paracellular permeability and maintaining cellular polarity (17–22). TJs comprise transmembrane proteins—such as claudins (which form the primary seal), occludin, and junctional adhesion molecules (JAMs)—that are linked to the actin cytoskeleton via intracellular scaffold proteins like ZO-1 (23, 24). While the core components of TJs are conserved across epithelia, the specific claudin family members expressed confer tissue-specific properties. For instance, barrier-forming claudins (Claudin-1, -3, -4, -8) are crucial in both airway and skin epithelium, whereas the expression of pore-forming claudins (Claudin-2, implicated in paracellular water and ion flow) is more prominent in “leaky” epithelia like the intestine. Underlying the TJs, adherens junctions (dependent on E-cadherin) provide strong mechanical adhesion and play a key role in initiating and stabilizing cell-cell contacts (17, 19, 20, 24). Desmosomes confer additional structural resilience by tethering intermediate filaments to the plasma membrane, a feature particularly critical in mechanically stressed tissues such as the skin (25–28) (Figure 2).

#### 3.1.2 The chemical and functional barrier

Beyond the physical seal, the airway epithelium possesses sophisticated chemical and functional defense systems. It is equipped with a mucociliary clearance apparatus: goblet cells secrete a mucus layer that traps inhaled particles, allergens, and pathogens, while the coordinated beating of cilia propels this mucus towards the pharynx for expulsion (4–6). This system, while analogous to the perpetual flow of intestinal contents, is uniquely adapted for the air-facing surface of the airways. Furthermore, airway epithelial cells secrete a plethora of antimicrobial peptides (AMPs)—such as defensins and cathelicidins—and other immune mediators like surfactant proteins A and D, which directly neutralize microbes and modulate immune responses (29). The production of secretory IgA, a key immune mediator at mucosal surfaces, is a shared feature of both the respiratory and intestinal tracts, although its induction occurs through distinct regional immune tissues (30–32).

#### 3.1.3 The immunological barrier

The epithelium functions as an active sentinel for the immune system. It expresses a wide array of pattern recognition receptors (PRRs), including Toll-like receptors (TLRs) and NOD-like receptors (NLRs), which detect conserved microbial motifs known as pathogen-associated molecular patterns (PAMPs) (33–35). Upon activation, epithelial cells produce not only AMPs and mucus but also release cytokines and chemokines that recruit and instruct innate and adaptive immune cells. This sentinel function is conserved across different barrier tissues; however, the specific immune cell populations recruited and the subsequent immune response profile are highly dependent on the tissue context (36, 37). A critical shared function of barriers, particularly the airway and intestine, is the promotion of immune tolerance to innocuous environmental and commensal antigens, a process involving complex interactions with resident dendritic cells and regulatory T cells (Tregs).

In summary, the airway epithelial barrier is a multifunctional and dynamic interface. Its core defensive mechanisms—physical intercellular junctions, mucociliary and chemical clearance, and active immune surveillance—are principles shared with other barriers like the gut and skin. However, its specific cellular composition, the mucociliary escalator, and the unique immunological milieu of the respiratory tract define its distinct role in health and disease.

### 3.2 Epidemiological and environmental factors influencing epithelial barrier dysfunction in allergic airway diseases

#### 3.2.1 Epidemiological characteristics of allergic respiratory diseases

The prevalence of allergic respiratory diseases exhibits distinct epidemiological patterns globally (38). Significant geographical variations exist; for instance, pollen sensitization is more common across European nations, whereas house dust mite allergy predominates in regions characterized by high humidity levels (39). Age is a critical determinant, with children and adolescents demonstrating the highest incidence rates (40). While symptom amelioration may occur with age in some individuals, others experience persistent or progressively worsening disease. Gender differences have also been documented, with males showing a higher predisposition to asthma during childhood, whereas a female predominance emerges in adulthood. Furthermore, genetic predisposition plays a fundamental role, as a positive family history of atopy substantially increases individual susceptibility (41). The economic burden associated with these conditions is considerable. A comprehensive study in Italy estimated the total economic cost—encompassing both direct medical expenses and indirect costs such as productivity losses—to be approximately €7.33 billion, with direct costs accounting for 72.5% and indirect costs for 27.5% of this total (42).

#### 3.2.2 Impact of environmental factors on the epithelial barrier and allergic diseases

Environmental factors significantly contribute to the pathogenesis of allergic respiratory diseases by directly compromising epithelial barrier integrity and promoting immune dysregulation. Air pollution, comprising particulate matter (PM2.5, PM10) and gaseous components, represents a major risk factor. Chronic exposure to polluted environments induces epithelial damage, downregulates the expression of tight junction proteins (claudins, occludin), and consequently increases barrier permeability. A pertinent example involves diesel exhaust particles (DEPs), which have been shown to disrupt tight junctions in nasal epithelial cells, thereby enhancing allergen penetration and exacerbating the symptoms of allergic rhinitis (Table 1).

**Table 1:** Environmental factors, molecular targets, and their impact on epithelial barrier integrity.

Environmental factor	Representative agent/change	Primary molecular target/mechanism	Impact on epithelial barrier
Air Pollutants	Diesel Exhaust Particles (DEPs)	Induction of oxidative stress (ROS); activation of the aryl hydrocarbon receptor (AhR)	Downregulates expression of tight junction proteins (e.g., occludin, ZO-1), disrupts their structure, increases permeability
	Particulate Matter (PM2.5/PM10)	Induction of oxidative stress; direct physical damage	Induces cell death, compromises barrier integrity; promotes the release of alarmins (TSLP, IL-33)
Allergens	House Dust Mite (Der p 1)	Protease activity directly cleaves tight junction proteins	Directly disrupts barrier structure, facilitates allergen penetration
Climatic Factors	Rising Temperatures, Elevated CO_2_ Levels	Alters the distribution, concentration, and allergenicity of aeroallergens	Prolongs exposure duration, increases barrier burden; certain pollens themselves can induce oxidative stress
Lifestyle Factors	Westernized Diet	Alters gut microbiota; influences systemic immunity via the gut-lung axis	Indirectly impairs respiratory barrier function and immune regulation; increases systemic inflammation

Climate change further modulates the prevalence and severity of allergic diseases. Rising global temperatures, altered precipitation and humidity patterns, and an increased frequency of extreme weather events collectively influence the distribution, seasonality, and allergenicity of aeroallergens. Notably, extended pollen seasons and increased pollen production, driven by warming climates, contribute to heightened sensitization rates and a greater symptom burden in affected populations. Moreover, climate-related shifts in microbial composition may disrupt epithelial homeostasis and immune balance, thereby facilitating the development of allergic responses.

Modern lifestyle changes also contribute to epithelial barrier impairment and increased allergic susceptibility. Westernized diets, characterized by high intake of saturated fats, refined sugars, and processed foods coupled with low dietary fiber content, may induce gut dysbiosis and compromise intestinal barrier integrity. This dysfunction can, in turn, perturb systemic immune regulation via the gut-lung axis, thereby elevating the risk of respiratory allergies.

### 3.3 Pathological mechanisms of the epithelial barrier in allergic airway diseases

#### 3.3.1 Regulatory mechanisms of the epithelial barrier in immune defense

**Figure 3:**
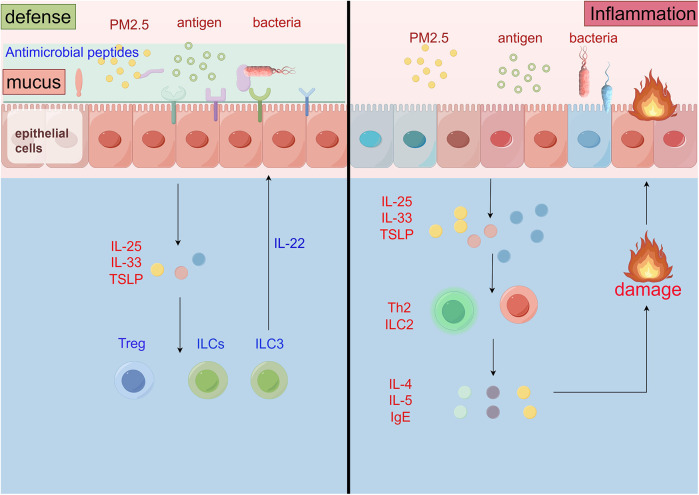
Pathological mechanisms of the epithelial barrier in allergic airway diseases.

The epithelial barrier effectively maintains mucosal immune homeostasis through multiple coordinated mechanisms. Physically, epithelial cells form continuous and dynamic defense structures via tight junctions (TJMs), adherent junctions (AJs), and desmosomes. Tight junction proteins such as claudins, occludin, and ZO-1 play crucial roles in regulating paracellular permeability, limiting cross-epithelial transfer of allergens and pathogens. Chemically, the mucus layer secreted by epithelial cells covers the mucosal surface, containing antimicrobial peptides, IgA, and surfactant proteins that directly neutralize or kill invading microorganisms. Additionally, epithelial cells express pattern recognition receptors (PRRs) including Toll-like receptors (TLRs) and NOD-like receptors (NLRs), which identify microbial-associated molecular patterns (MAMPs) to initiate innate immune responses. Immunomodulatory aspects include epithelium-derived cytokines like thymic matrix lymphocyte factor (TSLP), IL-25, and IL-33, which activate type II innate lymphocytes (ILCs) and regulate Th2-type immune responses (43), playing key roles in maintaining mucosal stability and moderate inflammation. Notably, intestinal and respiratory epithelial tissues further maintain immunological tolerance by regulating the differentiation and function of regulatory T cells (Tregs) (44) (Figure 3).

#### 3.3.2 The epithelial barrier as an inflammatory amplifier: underlying mechanisms

Beyond its role as a passive defensive structure, the epithelial barrier can actively amplify inflammatory responses under pathological conditions, thereby exacerbating allergic airway diseases. Upon exposure to allergens or following barrier injury, epithelial cells release a suite of alarmins and pro-inflammatory cytokines—including IL-33, IL-25, and TSLP—that activate ILC2s and Th2 lymphocytes (45). This activation enhances type 2 immune responses, leading to intensified inflammation. For instance, in allergic rhinitis, proteolytic allergens such as those from house dust mites stimulate nasal epithelial cells to release IL-33, which in turn promotes ILC2 and Th2 activation and the secretion of IL-5 and IL-13, resulting in eosinophil infiltration, mucus overproduction, and heightened symptom severity (46).

Barrier impairment also facilitates the invasion of pathogens such as bacteria and viruses, which can further propagate inflammatory cascades. Pathogen recognition by epithelial cells activates intracellular signaling pathways—notably NF-κB—leading to enhanced production of cytokines and chemokines that recruit additional immune cells to the site of inflammation, thereby amplifying the response. Concurrently, infection-induced epithelial cell death results in the release of damage-associated molecular patterns (DAMPs), such as high-mobility group box 1 (HMGB1), which further activate immune cells and intensify the inflammatory milieu (47).

Furthermore, bidirectional crosstalk between epithelial and immune cells contributes to inflammatory amplification. Epithelial expression of co-stimulatory molecules enhances T cell activation and proliferation, promoting increased cytokine production and sustained inflammation. In asthma, interactions between airway epithelial cells and Th2 cells reinforce type 2 cytokine secretion, worsening airway inflammation and hyperresponsiveness (48).

The epithelial barrier also plays a central role in activating both ILC2s and Th17 cells, thereby shaping different endotypes of allergic airway disease. When epithelial cells are damaged by allergens, pollutants, or injury, they rapidly release alarmins. IL-33 acts as a key signal, directly activating mucosal ILC2s and driving their production of large quantities of IL-5 and IL-13. This mechanism operates independently of adaptive immunity, rapidly initiating eosinophil infiltration, mucus hypersecretion, and airway hyperresponsiveness. Simultaneously, epithelial-derived cytokines such as TSLP and IL-1β can promote dendritic cell-mediated differentiation of Th17 cells (49). Th17-derived IL-17A subsequently acts on the epithelium to induce the production of chemokines like IL-8, recruiting neutrophils—a process closely associated with neutrophilic inflammation in severe and steroid-resistant asthma. Consequently, the epithelium, by differentially activating the ILC2/Th2 and Th17 pathways, not only amplifies type 2 inflammation but also drives disease heterogeneity and the formation of distinct inflammatory endotypes (Figure 2).

#### 3.3.3 Cytokine–epithelial barrier interactions in allergic inflammation

A complex and dynamic interplay exists between cytokines and the epithelial barrier, playing a pivotal role in the pathogenesis and progression of allergic respiratory diseases. Upon exposure to pathogens, allergens, or other inflammatory stimuli, epithelial cells secrete an array of cytokines—including IL-6, IL-8, and IL-33—that recruit and activate immune cells, thereby modulating both innate and adaptive immune responses (50, 51). For example, IL-8 acts as a potent chemoattractant for neutrophils and eosinophils; while essential for host defense, its overproduction can exacerbate inflammatory damage in allergic settings (50).

Conversely, cytokines derived from immune cells significantly influence epithelial barrier integrity. Th2-type cytokines, particularly IL-4and IL-13, have been shown to downregulate the expression of tight junction proteins, thereby increasing paracellular permeability and facilitating allergen penetration. In asthma, IL-13-mediated disruption of barrier function contributes to disease persistence and severity (44).

Beyond their pro-inflammatory effects, certain cytokines contribute to epithelial repair and homeostasis. Keratinocyte growth factor (KGF) promotes the proliferation and migration of epithelial cells, supporting barrier regeneration (52). In inflammatory bowel disease, KGF enhances mucosal healing and restores epithelial integrity (52). Similarly, in allergic airways, strategies aimed at modulating cytokine networks may offer therapeutic potential by promoting barrier restoration and suppressing inflammation.

### 3.4 Diagnostic techniques for the epithelial barrier in allergic respiratory diseases

#### 3.4.1 Novel approaches for assessing epithelial barrier function

Conventional methods for assessing epithelial barrier function have primarily relied on measuring transepithelial electrical resistance (TEER) and paracellular permeability. However, these techniques present limitations in capturing the complex physiological state of the barrier. Recent technological advances have introduced multiple novel assessment approaches to this field.

At the molecular level, proteomic and metabolomic technologies enable comprehensive analysis of global changes in proteins and metabolites within epithelial cells. These methods reveal molecular expression profiles related to tight junction and cytoskeletal regulation, offering fresh perspectives for understanding barrier function. For morphological observation, techniques like confocal laser scanning microscopy facilitate high-resolution, real-time monitoring of epithelial cell morphology and protein distribution (53). For instance, examining the localization of tight junction proteins in intestinal epithelial tissue allows direct evaluation of barrier structural integrity (53). Additionally, nanoparticle-based probe technology, which detects molecular changes on the epithelial surface, provides a novel sensitive method for barrier function assessment (54).

Regarding model systems, advancements in *in vitro* culture techniques have significantly improved physiological relevance in research. Air-liquid interface (ALI) cultures of respiratory epithelial cells (using lines such as Calu-3 and BEAS-2B) effectively mimic the structure and function of *in vivo* airway epithelium, making them suitable for studying environmental impacts on the barrier (55). The intestinal-derived Caco-2 cell line, capable of forming high-resistance tight junctions, has also become a standard model for barrier function studies. More sophisticated co-culture systems, which combine epithelial cells with immune cells like THP-1-derived macrophages, establish experimental platforms that better replicate physiological conditions (55). These systems provide powerful tools for investigating interactions between epithelial and immune systems.

#### 3.4.2 Research on biomarkers in allergic respiratory diseases

The identification of reliable biomarkers is crucial for the early diagnosis, disease monitoring, and personalized treatment of allergic respiratory diseases. Serum total IgE and allergen-specific IgE remain cornerstone biomarkers for diagnosing allergic sensitization (56). Elevated levels of total IgE are commonly associated with allergic conditions such as allergic rhinitis and asthma, while specific IgE testing helps identify relevant allergens and guides allergen-specific immunotherapy (56).

Fractional exhaled nitric oxide (FeNO) has emerged as a non-invasive biomarker reflecting eosinophilic airway inflammation. FeNO levels are frequently elevated in patients with asthma and correlate with the degree of type 2 inflammation (56). It serves as a useful tool for assessing asthma control, predicting exacerbations, and monitoring response to anti-inflammatory treatments.

Additionally, several cytokines and inflammation-related proteins show promise as biomarkers. Periostin, a matricellular protein induced by IL-4 and IL-13, is associated with Th2-mediated inflammation (56). Elevated serum periostin levels correlate with asthma severity and subphenotypes such as eosinophilic inflammation. Recent studies suggest that reductions in periostin following house dust mite sublingual immunotherapy are associated with improved lung function, highlighting its potential role in evaluating treatment response (56).

#### 3.4.3 Advances in imaging techniques for epithelial barrier research

Imaging technologies play an essential role in the study of the epithelial barrier, providing high-resolution tools to assess both structural and functional aspects of barrier integrity under physiological and pathological conditions. While conventional imaging modalities such as x-ray and computed tomography (CT) are useful for visualizing gross anatomical changes, their resolution is insufficient for analyzing the microscopic architecture and dynamic alterations of the epithelial barrier.

Advanced high-resolution microscopic techniques have significantly enhanced our ability to examine epithelial ultrastructure. Confocal laser scanning microscopy (CLSM) and two-photon microscopy (TPM) enable detailed real-time visualization of tight junction distribution, cytoskeletal organization, and cellular morphology (53). For instance, CLSM imaging of nasal epithelial cells has been used to document allergen-induced disorganization of ZO-1, providing direct evidence of barrier impairment in allergic rhinitis (53).

Magnetic resonance imaging (MRI)-based techniques are also being adapted for the functional assessment of barrier integrity. Diffusion-weighted imaging (DWI), which measures water molecule diffusion in tissues, can indirectly reflect epithelial permeability and has been applied in studies of intestinal inflammatory diseases to evaluate the extent of barrier damage (55). Optical coherence tomography (OCT), offering non-invasive, high-resolution cross-sectional imaging, is increasingly used in respiratory research to evaluate epithelial thickness, mucosal integrity, and remodeling processes. In asthma, OCT allows longitudinal monitoring of airway epithelial changes, providing valuable insights into disease progression and therapeutic responses (Table 2).

**Table 2:** Comparison of diagnostic techniques for assessing epithelial barrier integrity.

Technique category	Specific methods	Advantages	Limitations	Primary application context
Functional Assessment	Transepithelial Electrical Resistance (TEER)	Real-time, quantitative, non-destructive; Gold standard method	Primarily suitable for *in vitro* models; Lacks spatial information	Basic research, drug screening
	Paracellular Permeability Assay (e.g., FITC-Dextran)	Simple, intuitive, low cost	Mostly an end-point assay; Lacks real-time dynamic data	Basic research, barrier function validation
Imaging Techniques	Confocal Laser Scanning Microscopy (CLSM)	High resolution; Enables localization and semi-quantification of specific proteins (ZO-1)	Samples require fixation; Not suitable for long-term *in vivo* observation	Basic research, mechanistic investigation
	Optical Coherence Tomography (OCT)	Non-invasive; Enables *in vivo* longitudinal monitoring of airways	Limited molecular specificity; Resolution lower than microscopy	Clinical research, assessment of airway remodeling
	Diffusion-Weighted Magnetic Resonance Imaging (DWI-MRI)	Non-invasive; Can assess tissue permeability throughout the body	Relatively low spatial resolution; Application in airways is exploratory	Clinical research (intestinal barrier)
Omics Analysis	Transcriptomics/Proteomics	Unbiased discovery of novel biomarkers and pathways	High cost; Complex data analysis; Far from clinical translation	Biomarker discovery, mechanistic research
*In vitro* Models	Air-Liquid Interface (ALI) Culture	Highly mimics *in vivo* airway physiology and differentiation	Long culture period, technically challenging	Mechanistic research, toxicology assessment
	Airway Organoids	Retains tissue architecture and cellular heterogeneity	High cost; Standardized protocols need establishment	Disease modeling, personalized medicine

### 3.5 Therapeutic strategies and interventions

#### 3.5.1 Advances in pharmacotherapy for epithelial barrier repair

Targeting epithelial barrier repair represents a crucial therapeutic direction for allergic respiratory diseases. Several pharmacological agents have demonstrated potential in promoting barrier restoration. Antioxidants, such as N-acetyl-L-cysteine (NAC), can mitigate oxidative stress-induced epithelial damage. NAC provides thiol groups that bolster intracellular antioxidant defenses, thereby reducing oxidative injury, promoting the expression of tight junction proteins, and consequently improving barrier function. Animal studies have shown that NAC treatment alleviates diesel exhaust particle-induced nasal epithelial barrier impairment and restores the expression of the tight junction protein ZO-1 (57).

Natural compounds derived from traditional medicines also exhibit barrier-repairing properties. Bioactive components like berberine and curcumin possess anti-inflammatory and antioxidant activities. These compounds modulate cell signaling pathways to stimulate epithelial proliferation and differentiation, thereby strengthening tight junctions. Artesunate can improve TH2 inflammatory response and epithelial injury by regulating sting signaling pathway (58).

Furthermore, growth factors are under investigation for their reparative potential. Keratinocyte growth factor (KGF) specifically promotes epithelial cell proliferation and migration, accelerating barrier restoration. In models of respiratory disease, KGF administration facilitates the repair and regeneration of airway epithelium, improves barrier function, and reduces inflammatory responses (52).

#### 3.5.2 Immunomodulatory therapies in allergic diseases

Immunomodulatory therapy represents a key strategy for managing allergic respiratory diseases by modulating the host immune response and attenuating allergic inflammation. Among these approaches, allergen-specific immunotherapy (AIT) remains the only treatment capable of altering the natural course of allergic disease. AIT involves the administration of gradually increasing doses of allergen extracts to induce immune tolerance and reduce allergic symptoms. In patients with allergic rhinitis and asthma, subcutaneous or sublingual AIT can shift the immune response from a Th2- to a Th1-dominated profile, decrease IgE production, and enhance the number and function of regulatory T cells (Tregs), thereby alleviating allergic inflammation.

Other immunomodulatory agents are also employed. Vitamin D exhibits immunomodulatory effects by influencing the function of T lymphocytes and dendritic cells, suppressing Th2-type responses, and reducing allergic inflammation. Although vitamin D deficiency is associated with an increased risk of allergic disorders, the therapeutic efficacy of vitamin D supplementation remains controversial and warrants further investigation.

Probiotics are another area of interest, with evidence suggesting they may modulate immune function through interactions with the gut microbiota. Specific strains, such as Lactobacillus rhamnosus GG, have been shown to regulate intestinal immune responses, reduce Th2 cytokine secretion, and ameliorate allergic symptoms. However, further studies are needed to clarify their efficacy and appropriate target populations.

#### 3.5.3 Impact of novel biological agents on the epithelial barrier

The development of novel biological agents has introduced promising therapeutic avenues for allergic respiratory diseases, with increasing evidence supporting their beneficial effects on epithelial barrier function. Monoclonal antibodies such as anti-IgE (omalizumab), anti-IL-5 (mepolizumab, reslizumab), and anti-IL-4Rα/IL-13 (dupilumab) ameliorate allergic inflammation by specifically targeting key molecules within the type 2 immune pathway. By reducing eosinophilic inflammation and dampening immune activation, these biologics may indirectly promote epithelial repair and enhance barrier integrity. For instance, omalizumab has been shown to reduce airway inflammation and epithelial injury in asthma, suggesting a potential role in preserving barrier function (Table 3).

**Table 3:** Evaluation of therapeutic strategies targeting the epithelial barrier.

Therapeutic strategy	Representative agents/methods	Mechanism of action	Efficacy evidence level	Potential side effects/limitations
Direct Repair	N-Acetylcysteine (NAC)	Provides glutathione precursor, reduces oxidative stress, promotes tight junction protein expression	Supported by preclinical and some clinical studies	Relatively mild efficacy, requires long-term use
	Berberine, Curcumin, Artesunate	Anti-inflammatory, antioxidant; promotes barrier repair via modulating multiple signaling pathways	Primarily evidence from basic research	Low oral bioavailability, lack of large-scale clinical trials
	Keratinocyte Growth Factor (KGF)	Directly stimulates epithelial cell proliferation and migration	Strong preclinical evidence	Potential pro-tumor risk; safety requires evaluation
Immunomodulation	Allergen-Specific Immunotherapy (AIT)	Induces immune tolerance, may indirectly stabilize barrier function	Extensive clinical trials (Gold standard)	Long treatment course, risk of inducing allergic reactions
	Vitamin D	Modulates T-cell function, suppresses Th2 responses	Strong epidemiological association, inconsistent clinical trial results	Efficacy of supplementation remains controversial
Biologics	Omalizumab (anti-IgE)	Reduces free IgE levels, decreases mast cell/basophil activation, indirectly reduces epithelial injury	Extensive clinical trials (Marketed)	High cost, requires subcutaneous injection
	Dupilumab (anti-IL-4R*α*)	Dual blockade of IL-4/IL-13 signaling, reverses their induced barrier damage	Extensive clinical trials (Marketed)	Injection site reactions, ocular symptoms (e.g., conjunctivitis)
Emerging Therapies	Mesenchymal Stem Cells (MSCs)	Secretes various trophic factors and extracellular vesicles, exerts immunomodulatory and tissue-repair effects	Preclinical stage, promising prospects	Safety, standardization, optimal delivery route need determination
	Targeted siRNA	Precisely silences key genes causing barrier dysfunction (e.g., specific inflammatory factors)	Preclinical exploration stage	Challenges in delivery systems, long-term safety unknown

Cell-based therapeutic approaches are also under investigation. Mesenchymal stem cells (MSCs), known for their immunomodulatory and tissue-repairing properties, secrete a range of cytokines and growth factors that support epithelial proliferation and restoration. In experimental models of asthma, MSC administration attenuated lung inflammation, enhanced regeneration of airway epithelial cells, and strengthened barrier function, indicating therapeutic potential for respiratory disorders.

Moreover, emerging biologics such as small interfering RNA (siRNA) are being explored for their ability to modulate specific gene expression related to allergic inflammation and barrier dysfunction. siRNA constructs targeting pro-inflammatory cytokines or tight junction proteins offer a precision medicine approach to suppress inflammation and facilitate barrier recovery, though this strategy remains largely in the preclinical stage.

### 3.6 Controversies and future prospects

#### 3.6.1 The dual role of the epithelial barrier in allergic diseases

The role of the epithelial barrier in allergic diseases is characterized by a context-dependent duality, presenting a significant area of scientific inquiry. When intact, the epithelial barrier serves a protective function by preventing allergen penetration and maintaining immune tolerance through robust tight junctions and the secretion of antimicrobial agents (59). Conversely, barrier dysfunction—often marked by reduced tight junction protein expression—facilitates allergen translocation and the release of alarmins, such as IL-33 and IL-25. These alarmins activate ILC2s and Th2 cells, thereby initiating and amplifying type 2 inflammation (48). For instance, in allergic rhinitis, proteolytic allergens from house dust mites can directly compromise nasal epithelial tight junctions, increasing permeability and triggering a robust Th2 immune response that exacerbates symptoms (60). This duality underscores the need to better understand the precise molecular mechanisms governing the shift from epithelial homeostasis to a pro-inflammatory state. The central controversy lies in fully elucidating the signaling networks and environmental cues that determine whether the epithelium acts as a defender of tolerance or a driver of pathology.

#### 3.6.2 Emerging technologies reshaping epithelial barrier research

Cutting-edge technologies are profoundly transforming research on epithelial barrier function and its role in disease. CRISPR-Cas9 genome editing enables precise manipulation of genes involved in tight junction formation and epithelial homeostasis, allowing for functional studies of barrier-related genes *in vitro* and *in vivo* (61). For example, generating knockout models of specific claudin genes in intestinal organoids has been instrumental in defining their unique contributions to paracellular sealing and pore formation (61).

Single-cell RNA sequencing (scRNA-seq) unveils cellular heterogeneity and dynamic transcriptional changes in epithelial populations under physiological and allergic conditions, revealing novel subtypes and disease-specific signatures. In allergic airway disease, scRNA-seq has identified distinct epithelial cell states associated with type 2 inflammation and remodeling, offering potential new therapeutic targets (62).

Additionally, organ-on-a-chip and microfluidic systems provide biomimetic microenvironments that simulate tissue-level barrier function, enabling real-time analysis of barrier integrity, immune-epithelial crosstalk, and response to allergens or drugs in a human-relevant context A prominent application is the “lung-on-a-chip” model, which can be used to study the real-time dynamics of allergen-induced barrier disruption and the efficacy of barrier-protective agents. Together, these technologies offer unprecedented resolution for investigating epithelial barrier regulation, accelerating the identification of novel mechanisms and potential therapeutic targets (55).

#### 3.6.3 Future directions in the treatment of allergic respiratory diseases

The management of allergic respiratory diseases is moving toward precision medicine and personalized therapeutic strategies. Future efforts will focus on targeting specific molecular pathways—particularly those governing epithelial barrier integrity and immune-epithelial crosstalk—to enable more effective and mechanism-based interventions. This includes the development of small-molecule inhibitors targeting specific epithelial signaling pathways to directly promote barrier restoration, used alongside or in conjunction with immunomodulatory therapies (63).

Advances in biologics, cell-based therapies (e.g., MSCs), and novel delivery systems hold promise for restoring barrier function and modulating localized immune responses with greater specificity (64). Gene therapy approaches, though in earlier stages, represent a frontier for achieving long-term disease modification by directly correcting underlying epithelial defects.

Concurrently, environmental health measures and lifestyle interventions will play an essential role in primary and secondary prevention. This involves strategies to reduce exposure to airborne pollutants, allergens, and dietary factors known to compromise barrier function. Integrating multimodal strategies that combine targeted pharmacotherapy with environmental control and patient education will be crucial to improving long-term outcomes and enhancing the quality of life for affected individuals.

## 4 Conclusion

This review systematically elucidates the central role of the airway epithelial barrier as a “dual-regulator” in allergic respiratory diseases. In its defensive capacity, the barrier maintains immune homeostasis through tight junctions, chemical secretions, and active immunomodulation. Conversely, under pathological conditions—where the barrier is compromised by genetic predisposition, environmental factors, or proteolytic allergens—it transitions to an active promoter of inflammation by releasing alarmins such as TSLP, IL-33, and IL-25. These mediators initiate and amplify ILC2/Th2-driven type 2 immune responses, establishing a vicious cycle of inflammation and barrier disruption. Based on this mechanistic understanding, barrier-centric therapeutic strategies demonstrate significant potential. These include direct barrier repair using antioxidants and growth factors, indirect restoration via biologics and immunotherapies that modulate upstream immune signals, and future exploration of combination strategies integrating allergen-specific immunotherapy with barrier-protective agents. The field is increasingly moving toward precision medicine, leveraging advanced technologies such as single-cell sequencing and organoid models to uncover novel epithelial subpopulations and complex cellular crosstalk. The ultimate goal is to develop biomarkers based on individual barrier status to guide disease risk prediction, endotype stratification, and therapeutic decision-making. This paradigm shift from symptomatic treatment to targeted etiology-focused therapy and disease modification holds promise for achieving long-term disease control and potentially paving the way for a cure, offering new hope for patients affected by these conditions.
